# Why can’t I see after my heart is fixed: a case series of ocular complications after cardiac intervention

**DOI:** 10.1186/s12886-016-0209-1

**Published:** 2016-03-25

**Authors:** Yong Meng Hsien, Mushawiahti Mustapha, Jemaima Che Hamzah, Oteh Maskon, Choor Chee Ken, Che Hassan Hamat Hamdi

**Affiliations:** Department of Ophthalmology, Universiti Kebangsaan Malaysia Medical Centre, 56000 Cheras, Kuala Lumpur, Malaysia; Unit of Cardiology, Department of Medicine, Universiti Kebangsaan Malaysia Medical Centre, 56000 Cheras, Kuala Lumpur, Malaysia

**Keywords:** Retinal artery occlusion, Percutaneous coronary intervention, Coronary artery disease, Acute coronary syndrome, Thromboembolism

## Abstract

**Background:**

The purpose of this study was to report case series of retinal artery occlusion (RAO) as one of the significant complication post cardiac intervention.

**Case presentation:**

We are reporting one case of branch RAO and one case of central RAO after percutaneous coronary intervention (PCI). In case 1, a 74-year-old gentleman with pre-existing diabetes mellitus and hypertension was electively admitted for PCI for coronary artery disease (CAD) in our centre (UKMMC). Few hours after the procedure, patient complained of sudden blurring of vision in the right eye. He was found to have branch retinal artery occlusion. In case 2, a 49-year-old gentleman presented with ST segment elevation myocardial infarction (STEMI) and had an emergency PCI performed 2 h upon admission. He noticed sudden dropped of vision in his right eye immediately after the procedure. He was diagnosed to have central retinal artery occlusion.

**Conclusions:**

In conclusion, retinal artery occlusion is a possible complication post PCI. Patients need to be informed especially in high risk cases.

## Background

Percutaneous coronary intervention (PCI) or coronary angioplasty is a cardiac procedure to treat a significant coronary artery disease and as part of revascularisation to restore coronary artery flow and improved patients outcome. There are two access routes frequently described with regards the procedure: the transfemoral and transradial approaches. In acute setting, it is a life saving procedure. However some complications still could not be avoided [1]. This procedure is currently a standard procedure for both diagnostic and therapeutic purpose in management of coronary artery diseases (CAD).

Thromboembolic events are one of its major risks leading to end organ infarction, especially in the brain, kidney, and the eyes [1]. One of the possible thromboembolic events in the eyes is retinal artery occlusion, either branch (BRAO) or central (CRAO), which will lead to a wide spectrum of visual problems from asymptomatic, partial loss of vision to complete blindness. Therefore, there should be a high index of suspicion and immediate attention should be given to any visual disturbance in this group of patients.

To the best of our knowledge, since the introduction of PCI in 1977, case reports of retinal artery occlusion (CRAO & BRAO) post-PCI have comprised ten individual case reports from around the world, with the earliest being that in 1985 by Stefansson et al. [2], and the most recent being that in 2010 by Selton et al. [3]. Herein, we report two cases of retinal artery occlusion following PCI in our centre. Both cases were complicated by significant visual morbidity.

## Case presentations

### Case 1

A 74-year-old gentleman with coronary risk factors of diabetes mellitus and hypertension was electively admitted for PCI for CAD in UKMMC. Diagnostic coronary angiogram revealed severe CAD with stenosis at the bifurcation of the artery. Coronary angioplasty was performed using drug-eluting stent (DES) for right coronary artery (RCA), left main stem coronary artery (LMS) and left anterior descending artery (LAD). Good angiographic flow was obtained post stents dilatation. On top of the dual antiplatelet therapy (aspirin and clopidrogrel) started prior to the procedure, patient was loaded with clopidrogrel as well during the PCI. Electrocardiogram (ECG) showed sinus rhythm throughout and after the procedure. Bedside echocardiography showed good left ventricular ejection fraction without evidence of clot.

**Fig. 1:**
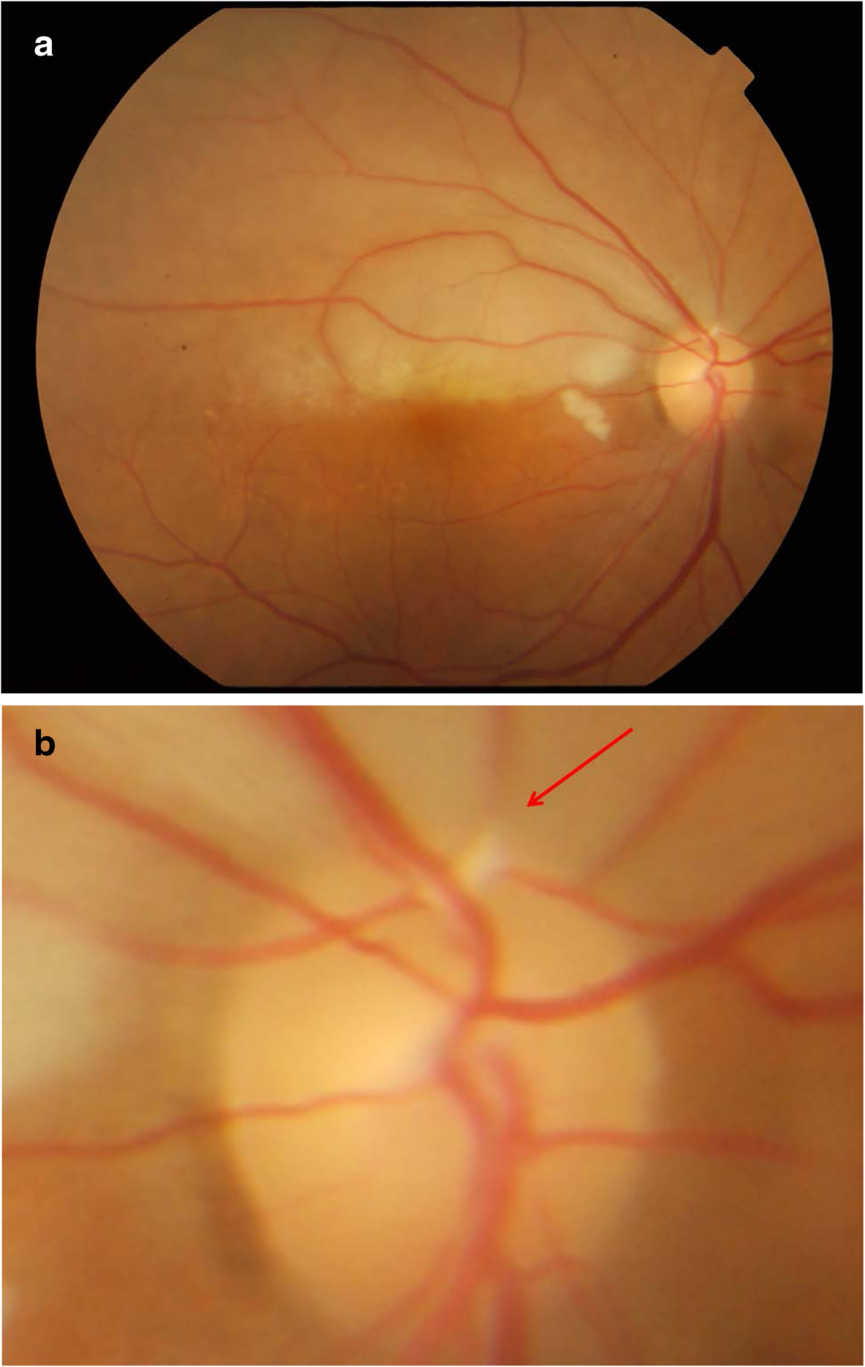
Case 1: Right eye findings. **a** Fundus photo showing superior pale retina. **b** Presence of Hollenhorst plaque in the superotemporal vessel (*arrow*)

However, immediately after it the patient complained of loss of vision in his right eye (RE), specifically involving the lower visual field. Visual acuity in the RE was 6/18 with an inferior visual hemifield defect. Fundus examination revealed BRAO involving the superior branches of the retinal artery with the presence of multiple emboli or Hollenhorst plaque in the branches of the superior retinal artery (Fig. 1a and b). The superior retina was swollen and pale (Fig. 1a). Complete neurological examination revealed no neurological deficit. No carotid bruit detected. ECG was stable without evidence of arrhythmia. Computed tomography (CT) scan of the brain was performed and no abnormalities were detected.

Despite immediate conventional treatment for his BRAO was given, his RE inferior hemi-field loss was persistent. The condition remained stable and patient was informed for the guarded prognosis for further visual field recovery. Case was decided to continue on dual antiplatelet therapy without heparin therapy. He was monitored in CRW for another 48 h and discharged with stable vital sign and no evidence of new thromboembolic event.

Dual antiplatelet was continued and planned for lifelong aspirin with one year clopidogrel by his cardiologist for his underlying treated coronary artery disese.

### Case 2

A 49-year-old gentleman with no past medical history presented to the emergency room of UKMMC with acute coronary syndrome. He was diagnosed with STEMI over the anterior leads. Primary PCI was carried out 2 h after and revealed 60–70 % blockage over the LAD and left ventricular clots by bedside echocardiogram. Angioplasty to LAD was done on the same setting. On day 2 of admission the case was further complicated with paroxysmal atrial fibrillation (AF) needed enoxaparin therapy which was converted to warfarin therapy later.

**Fig. 2:**
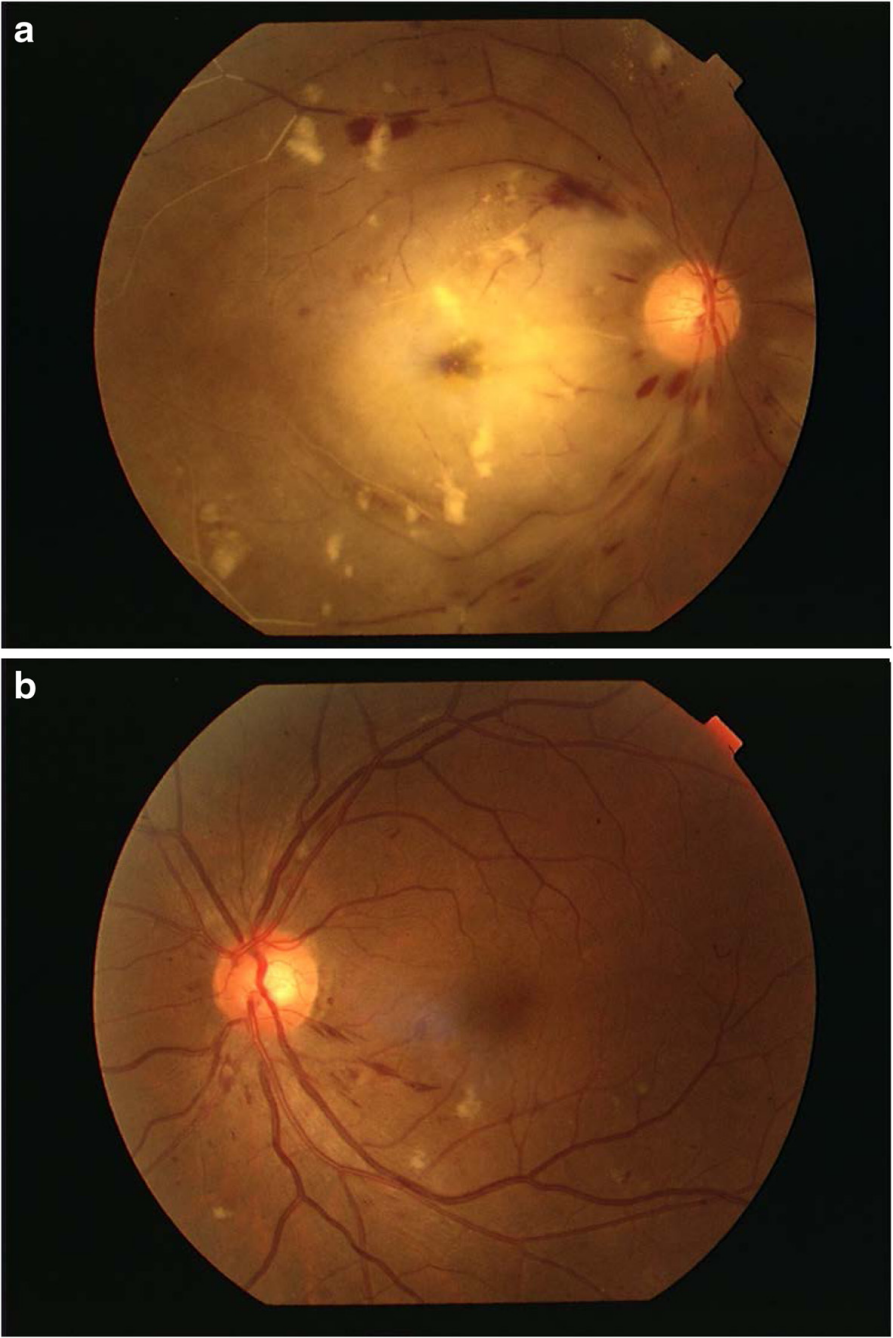
Case 2: **a** Right eye fundus showed extensive area of pale retina with cherry red spot. **b** Left eye fundus showed underlying diabetic retinopathy changes

The patient noted sudden loss of vision in his right eye immediately after the procedure but unfortunately he was too fatigued to complain to the treating doctor upon occurrence of the symptom. Only on day 6 of admission when patient was transferred to CRW, ophthalmological review noted that the patient was nonperceptive to light (NPL) over the RE with dense relative afferent pupillary defect (RAPD). There was evidence of CRAO with classical cherry-red spot in the macula with normal intraocular pressure. There were also associated with diabetic retinopathy (DR) changes bilaterally with dots-blots and flame-shaped haemorrhages which were undiagnosed prior to admission (Fig. 2a and b). Complete neurological examination and carotid examination were documented normal and CT brain showed no significant abnormality.

Due to the severe visual impairment (NPL), carotid Doppler ultrasound was done 3 weeks post PCI and revealed calcified plaque in the right proximal internal carotid artery (ICA). Magnetic resonance angiography (MRA) at 2 months post PCI revealed occlusion of almost the entire intracranial right ICA. Possible diagnosis of right ocular ischaemic syndrome (OIS) was also considered. However, during the acute presentation, there was no anterior segment involvement (normal IOP) with the presence of cherry red spot at the macula that denotes the possibility of preservation of the choroidal circulation during the acute setting. Both important features during the acute presentation were suggestive of CRAO rather than OIS. However, potentially continuous embolic event to the ocular blood supply might occur with time due to the severe stenotic damage involving the carotid artery as featured on the MRA. This possible continuous embolization might lead to full-blown ocular ischaemic syndrome. Repeat fluorescein angiography might be able to prove these changes. However it was not performed in this particular patient as retinal angiogram at this juncture lacks of any additional clinical therapeutic value. His right eye vision was NPL and did not improve. Subsequent follow-up 2 months after the PCI, he eventually developed rubeosis iridis which was treated with laser photocoagulation.

This patient was prescribed by his cardiologist with triple therapy with lifelong anticoagulant i.e. warfarin and aspirin and was also on clopidogrel for one month. Unfortunately patient was defaulted follow up 3 months post PCI.

## Discussion

Coronary angiogram with subsequent PCI is essentially a non-surgical procedure for diagnosis and treatment of coronary heart disease. This group of patients commonly present with stable angina, unstable angina or acute myocardiac infarction either STEMI or NSTEMI. Sometimes the failure symptoms also warrant this cardiac procedure. In addition to the underlying thromboembolic risk associated with CAD especially with associated arrhythmia, extension of left main stem artery (LMS), hypokinetic wall and also the present of intramural thrombus, PCI also poses a significant risk of embolization to other parts of the body including the ocular vessels. Furthermore, the procedure carries a mortality rate of up to 1.5 % [1].

The incidence of new retinal emboli post-PCI was 6.33 % (19 out of 300 subjects, all asymptomatic) in one landmark study [4]. The same study also found two conventional risk factors (age and hypertension) and operator expertise were strongly associated with new retinal emboli. On the other hand, the mode of the procedure (type of stent/duration) and severity of CAD had no effect on the risk of retinal emboli.

In both our cases immediate conventional treatment was applied due to delayed presentation, which included prompt ocular massage, reduction of intraocular pressure by intravenous acetazolamide, paracentesis, as well as carbon dioxide (CO2) rebreathing bag respiration. However, despite these immediate measures, vision outcome was poor in the CRAO case and remained unchanged in the BRAO case. The aim of treatment is reperfusion of the blocked retinal vessels especially when it is done within 6 h, since retinal ischemic damage after 240 min is usually irreversible. Reperfusion of blocked retinal vessels is believed to be able to reverse or reduce the damage caused by tissue infarction, only if it is done within a certain period of time limit, prior to permanent damage occuring. A study performed in monkeys showed that CRAO lasting for about 240 min resulted in massive and irreversible retinal damage [5]. There was no evidence of damage with CRAO of less than 97 min, but occlusion within 97–240 min produced a variable degree of damage.

The conventional treatment methods include ocular massage; reduction of intraocular pressure by various medical and surgical means; vasodilation of the CRA by sublingual isosorbide dinitrate and CO2 rebreathing bag; antiplatelet and heparin therapy. Other treatments reported with variable success include isovolemic hemodilution; hyperbaric oxygen; intravenous steroid to reduce vascular endothelial; Nd:YAG laser arteriotomy and embolectomy [6]; surgical embolectomy [7]; and intraarterial fibrinolysis delivered directly into the ophthalmic artery [8]. However, none of these methods are proven to be effective. Therefore, early diagnosis helps to identify the source of the problem, thus preventing further damage or recurrence to other important organ.

Computed tomography (CT) scan of the brain is indicated for the diagnosis of concurrent cerebral vascular event. This is one of the life threatening thromboembolic complications that needs to be ruled out. Moreover, cortical stroke itself can lead to poor vision without corresponding visual field and fundus changes. CT scan can help in ruling out these two conditions, either as the differential diagnosis or concurrent pathology.

## Conclusions

In conclusion, CRAO/BRAO is a possible complication post-PCI. The risk of CRAO/BRAO need to be discussed especially in high risk cases (ie, severe comorbidities, advanced age, associated arrhythmia, extension of left main stem artery (LMS), hypokinetic wall and also the presence of intramural thrombus). Prompt referral by the cardiologist and early treatment by ophthalmologists may help preserve or reduce the extent of damage by the occlusion.

### Consent

Written consent was obtained from all the patients for publication of this study.

## Abbreviations

PCI: percutaneous coronary intervention
CAD: coronary artery disease
STEMI: ST segment elevation myocardial infarction
CRAO: central retinal artery occlusion
BRAO: branch retinal artery occlusion
DES: drug-eluting stent
RCA: right coronary artery
LMS: left main stem coronary artery
LAD: left anterior descending artery
ECG: electrocardiogram
RE: right eye
CRW: cardiac rehabilitation ward
CT: computed tomography
AF: atrial fibrillation
RAPD: relative afferent pupillary defect
NPL: non-perceptive to light
DR: diabetic retinopathy
ICA: internal carotid artery
MRA: magnetic resonance angiography
OIS: ocular ischaemic syndrome
